# Impact of pre-operative edema index on surgical outcomes in supratentorial meningiomas: A retrospective analysis

**DOI:** 10.12669/pjms.41.13(PINS-NNOS).13365

**Published:** 2025-12

**Authors:** Usman Ahmad, Nabeel Sultan, Asad Iftikhar Shah, Syed Shahzad Hussain Shah

**Affiliations:** 1 Usman Ahmad, MBBS, FCPS. Neurosurgery Unit-II, Punjab Institute of Neurosciences, Lahore, Pakistan; 2 Nabeel Sultan, MBBS. Neurosurgery Unit-II, Punjab Institute of Neurosciences, Lahore, Pakistan; 3 Asad Iftikhar Shah, MBBS, MS. Neurosurgery Unit-II, Punjab Institute of Neurosciences, Lahore, Pakistan; 4 Prof. Dr. Syed Shahzad Hussain Shah, MBBS, FCPS. Neurosurgery Unit-II, Punjab Institute of Neurosciences, Lahore, Pakistan

**Keywords:** Edema Index, Peritumoral Brain Edema, Meningiomas, Resection

## Abstract

**Objective:**

To assess how pre-operative Edema Index (EI) affects surgical outcomes, complications, and hospital stay in patients with supratentorial meningiomas and its role as a predictor for surgical planning and prognosis.

**Methodology:**

A retrospective observational study was conducted at the Department of Neurosurgery Unit-II, Punjab Institute of Neurosciences, Lahore, over 14 months (January 2024–February 2025). 31 patients with supratentorial meningiomas were included through non-probability consecutive sampling.

**Results:**

Mean age was 41.39 years (range: 18–64 yrs), with male-to-female ratio of 1:1.82. 58.1% (18) were located in the convexity, followed by Parasagittal 12.9% (4), olfactory groove 6.5%(2) , parafalcine 6.5%(2), sphenoid wing 6.5%(2), tuberculum sellae 6.5%(2) and temporal 3.2%(1) regions. Based on size. 3.2% (1) of the tumors were small (<2cm), 35.5% (11) were medium (2-4cm), 38.7% (12) were large (4.1-6 cm) and 22.6% (7) were giant (>6cm). EI was <1 in 38.7% (12), 1–2 in 12.9% (4), and >2 in 32.3% (10); 16.1% (5) had no edema. Higher edema (EI >2) was more common in males (54.5%) than females (20%). Expansion duraplasty was required in 58.1% (18), bone removal in 9.7% (3) and bleeding occured in 6.5% (2). Grade I-II resections were achieved in 90% of patients with EI>2. Neurological deficits occurred in 6 (19.4%). 22.6%(7) had hospital stay exceeding 10 days.

**Conclusion:**

Pre-operative EI and tumor location significantly influences surgical complexity, extent of resection, complications, and hospital stay. Incorporating EI into preoperative evaluation can improve surgical planning and outcomes.

***Abbreviations:* PTBE:** Peritumoral brain edema, **EI**: Edema Index.

## INTRODUCTION

Supratentorial meningiomas are the most common primary intracranial tumors, accounting for a significant proportion of brain neoplasms.^1^, ^2^ These tumors arise from the meninges, the protective membranes surrounding the brain and spinal cord, and can occur in various regions of the brain, including the frontal, parietal, temporal lobes, and the convexity of the skull.^2^ Meningiomas are typically slow-growing and are usually benign, but their location and size can lead to substantial neurological impairments.^3^ The tumor’s proximity to critical brain structures, such as cranial nerves, blood vessels, and cortical areas, further complicates both diagnosis and treatment. Symptoms can vary significantly, depending on the tumor’s size and location, and may include focal neurological deficits, headaches, seizures, and visual disturbances. Some patients also experience cognitive dysfunction, adding to the complexity of management.^3^

The diagnosis of meningiomas is primarily based on radiological imaging, with magnetic resonance imaging (MRI) being considered the gold standard.^4^ MRI allows for detailed visualization of tumor location, size, and relationship to surrounding structures, which is essential for preoperative planning. However, diagnosing and managing meningiomas remains challenging, particularly due to their nonspecific clinical presentation and the potential for asymptomatic growth. By the time the symptoms are recognized, the tumor may already be of considerable size, further complicating treatment strategies.^5^ Surgical resection remains the most effective treatment for meningiomas, but the presence of peritumoral brain edema (PTBE) significantly complicates surgery.

PTBE refers to the accumulation of fluid in the brain surrounding the tumor and is frequently observed in meningiomas. It is associated with increased intracranial pressure, exacerbating neurological symptoms, and making tumor resection more difficult.^5^ The development of PTBE is influenced by several factors, including the vascular supply to the tumor, histological tumor subtype, and tumor-related venous obstruction.^6^,^7^ Tumors with abundant blood supply and those exhibiting aggressive growth patterns are often more likely to cause significant edema.^8^ Moreover, specific histological subtypes of meningiomas, such as those with a high vascular component or more aggressive cellular features, have been linked to a greater extent of PTBE.^8^

The vascular endothelial growth factor (VEGF-A) pathway has also been implicated in the development of PTBE, particularly in tumors with increased VEGF expression, which promotes vascular permeability and fluid leakage into the surrounding brain tissue.^9^-^11^ In addition, tumor-related venous obstruction can further exacerbate PTBE by impairing venous drainage and increasing pressure in the surrounding tissues, leading to further edema formation.^7^

Quantifying the severity of PTBE is important for predicting surgical complexity and clinical outcomes. The edema index (EI), calculated as the ratio of peritumoral edema volume to tumor volume, provides a valuable measure of the severity of PTBE.^5^ Tumors with a higher EI tend to be more difficult to resect, often requiring more extensive surgical procedures and posing greater risks for postoperative complications. Moreover, the presence of significant edema is often indicative of a more aggressive tumor with increased recurrence potential and poorer long-term outcomes.^5^ Despite its clinical relevance, the relationship between preoperative EI and long-term surgical and neurological outcomes has not been fully explored in the literature. Most studies have focused on short-term outcomes, such as surgical complications and immediate recovery times, leaving a gap in understanding how PTBE, as quantified by the edema index, influences long-term survival and neurological function.

This study aimed to investigate the prognostic significance of the edema index in patients with supratentorial meningiomas. By analyzing the correlation between preoperative EI and post-operative outcomes, the study seeks to provide insights into how the edema index can be used for preoperative planning and risk stratification. A better understanding of how PTBE, as measured by the EI, influences patient prognosis could lead to more personalized and effective treatment strategies. Early identification of patients with higher EI values could inform surgical approaches and postoperative care, potentially reducing the risk of complications and improving surgical outcomes. This research aimed to contribute to the growing body of literature on PTBE in meningioma management, ultimately enhancing clinical decision-making and improving patient care.

## METHODOLOGY

A retrospective observational study was conducted at Department of Neurosurgery Unit-II, Punjab Institute of Neurosciences, Lahore, over a period of 14 months (January 2024 to February 2025). This was a non-probability based consecutive case series of 31 patients fulfilling the following criteria.

### Ethical Approval:

This study was approved by the Institutional Review Board of Punjab Institute of Neurosciences, Lahore with reference no 2033/IRB/PINS/Approval/2025 dated January 23, 2025.

### Inclusion criteria:

- Patients between 18-65 years of age
- Both genders
- Diagnosed with supratentorial meningioma undergoing resection
- Pre-operative MRI-Brain available for all patients

### Exclusion criteria:

- Patients with recurrent meningiomas or non-supratentorial meningiomas
- Patients undergoing non-surgical treatments
- Incomplete pre-operative imaging or follow-up data

This study included 31 patients that underwent supratentorial meningioma resection. Their files and medical record was reviewed and data was collected in preformed Google forms. Data collected included the patient’s age, gender, location and size of the tumor, edema index, extent of resection, pre and post-operative complications. Tumor and edema volumes were measured using MRI (T2-weighted and contrast-enhanced T1 sequences). Manual segmentation was performed using RadiAnt Dicom Viewer software by two neurosurgeons independently.

### Data analysis technique:

The collected data were analyzed using a Microsoft Excel sheet. Continuous variables, such as age, were summarized with means and standard deviations, while categorical variables, including gender and location of the tumor, were presented as frequencies. Post-operative complications were depicted in bar graphs and frequencies

Data was analyzed by using Statistical Package for Social Sciences (SPSS) version 26. For stratification purposes age groups were compared with gender, location of lesion, size of lesion, and complications. P-values were calculated taking < 0.05 as significant.

## RESULTS

**Fig.1:**
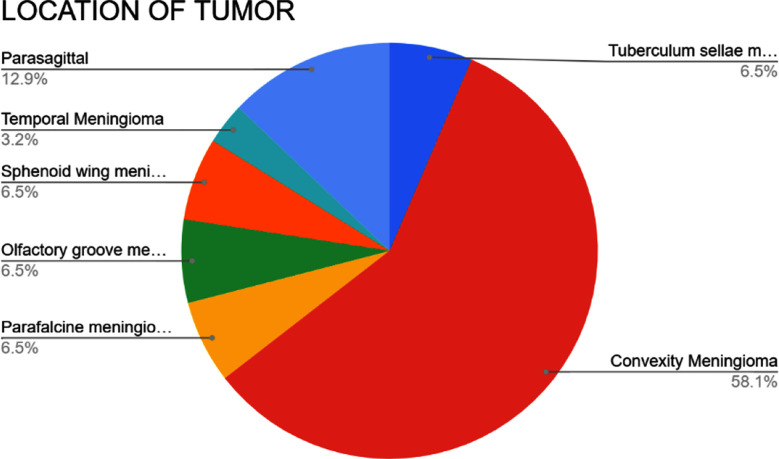
Pie chart illustrating the proportion of tumors by anatomical site. Convexity was the most common location (58.1%), followed by parasagittal (12.9%), skull base sites (olfactory groove, parafalcine, sphenoid wing, tuberculum sellae; 6.5% each), and temporal (3.2%).

A total of 31 patients with supratentorial meningiomas were included in this study. The mean age was 41.39 years (range: 18–64 years), with a male-to-female ratio of 1:1.82. Tumours were predominantly located in the convexity (58.1%), followed by 12.9% in parasagittal and 6.5% in olfactory groove, parafalcine, sphenoid wing and tuberculum sellae regions and 3.2% in temporal region (Fig.1).

***Size:*** tumors were put into four categories on the basis of size (Table-I).

- Small (<2cm) = 3.2%
- Medium (2-4cm) = 35.5%
- Large (4.1-6 cm) =38.7%
- Giant (>6cm) =22.6%

**Table-I:** categories summarizes the distribution of patients across different EI categories and their corresponding tumor sizes. Higher EI values were observed across all tumor size groups, but most frequently in large tumors.

Pre-operative Edema Index	No. of patients	Percentage	Size of tumor
0	5	16.1%	Small (<2cm) = 0 (0%)
			Medium (2-4cm) = 2 (6.45%)
			Large (4.1-6 cm) = 1 (3.2%)
			Giant (>6cm) = 2 (6.45%)
<1	12	38.7%	Small (<2cm) = 0 (0%)
			Medium (2-4cm) = 5 (16.12%)
			Large (4.1-6 cm) = 5 (16.12%)
			Giant (>6cm) = 2 (6.45%)
1-2	4	12.9%	Small (<2cm) = 0 (0%)
			Medium (2-4cm) = 1 (3.2%)
			Large (4.1-6 cm) = 1 (3.2%)
			Giant (>6cm) = 2 (6.45%)
>2	10	32.3%	Small (<2cm) = 1 (3.2%)
			Medium (2-4cm) = 3 (9.67%)
			Large (4.1-6 cm) = 5 (16.12%)
			Giant (>6cm) = 1 (3.2%)

The Pre-Operative Edema Index (EI) was classified into three groups, 16.1% of the cases had no edema, (Table-I).

- EI < 1: 38.7% of cases
- EI 1–2: 12.9% of cases
- EI > 2: 32.3% of cases

### Intraoperative and postoperative complications:

**Fig.2:**
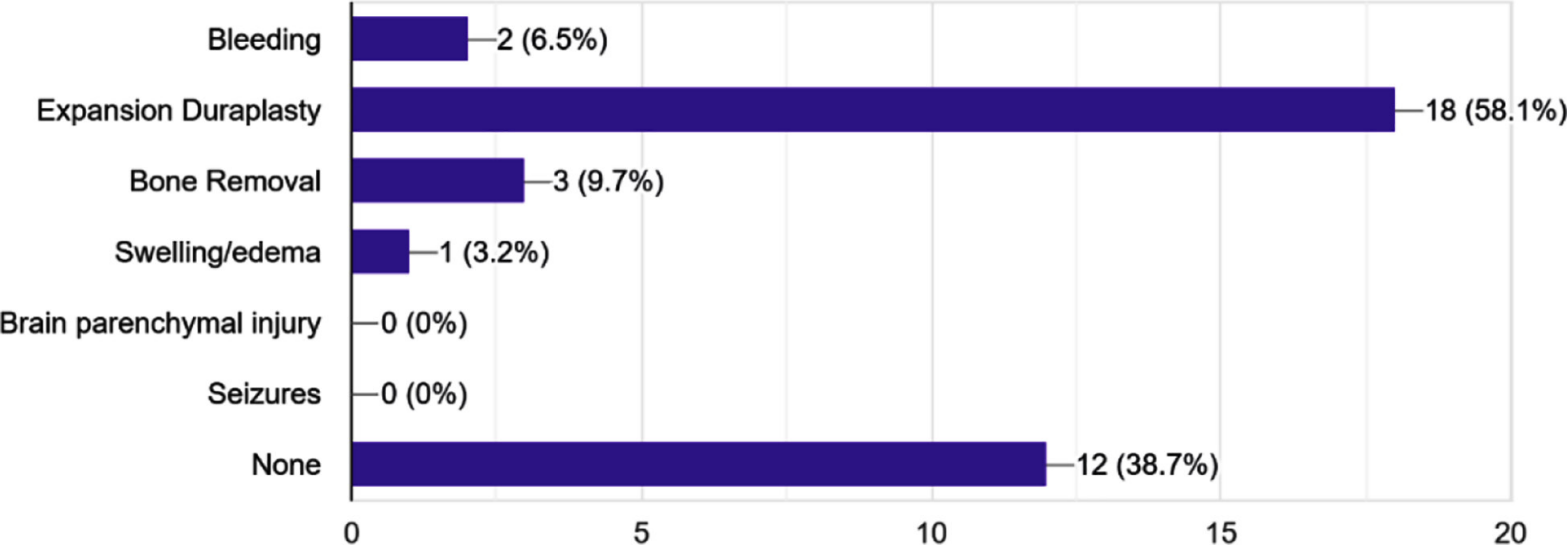
Bar graph showing frequency of intra-operative complications including expansion duraplasty, bone removal, intraoperative bleeding, and brain swelling.

Expansion duraplasty was required in 58.1% of cases. Out of the 10 patients with EI > 2, expansion duraplasty was done in seven. And out of four cases with EI= 1-2, 2 underwent expansion duraplasty. Expansion was also done for six out of the 12 cases with EI<1 and for three cases out of five with no edema. Bone removal was done in 9.7% of the patients (three cases). One with EI=0, one with EI<1 and one with EI=1-2. Bleeding was reported in 6.5%% (two cases) of the cases. One with EI=0 and one with EI<1. Intra-op swelling was also present in one case (Fig.2).

**Fig.3:**
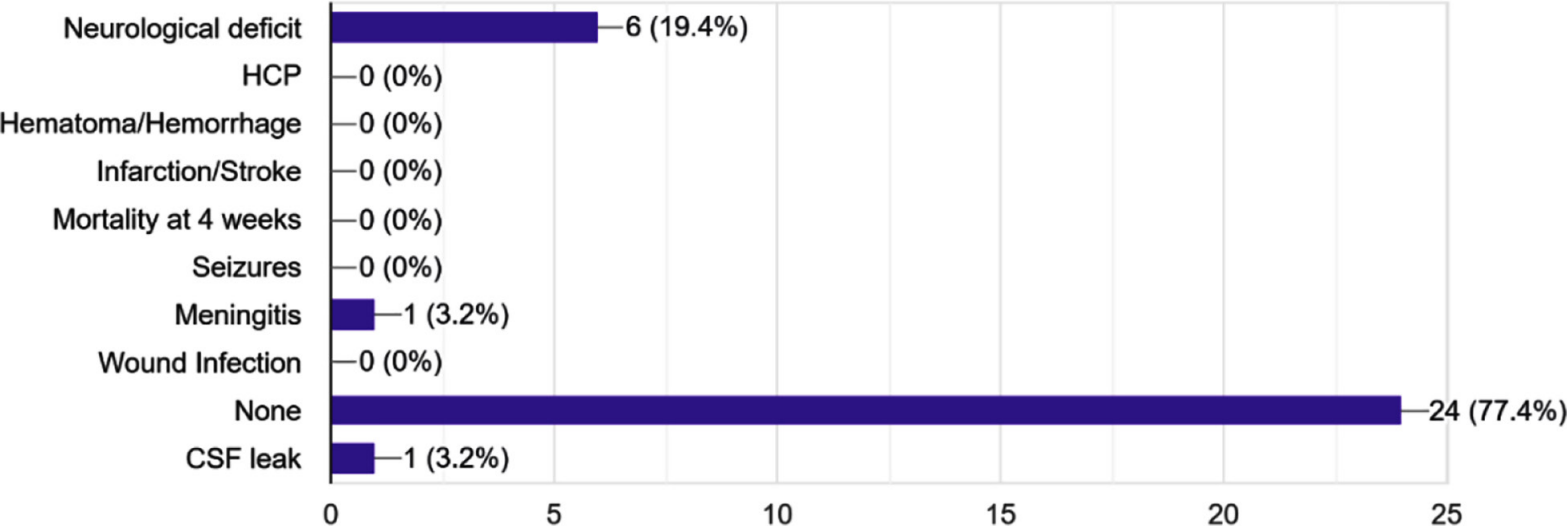
Bar graph showing frequency of complications including expansion duraplasty, bone removal, intraoperative bleeding, and brain swelling. Expansion duraplasty was most frequent in patients with EI

Contrary to initial expectations, the extent of resection was not significantly influenced by the edema index. Simpson Grade I-II resections were achieved at comparable rates across all EI groups, suggesting that while extensive peritumoral edema presents surgical challenges, it does not necessarily preclude achieving a gross total resection (GTR). Neurological deficits occurred in six patients (19.4%). Two with EI>2, 2 with EI=0, 1 with EI=1-2 and one with EI<1. Meningitis occurred in one case (EI>2) and CSF leak in one case with EI<1 (Fig.3).

### Postoperative hospital stay:

- The duration of post-operative hospitalization was directly influenced by the preoperative EI. 80% of the patients with EI>2 had stays of more than 5 days. In contrast 50% patients with EI=0 and EI<1 were discharged in the first 5 days after surgery (Table-II).
- Less than 5 days: 35.5% of patients (mostly EI < 1 and EI=0). 8 out of 11 patients discharged within 5 days of surgery fall in these two groups
- 5-10 days: 41.9% of patients
- More than 10 days: 22.6% of patients (equal distribution among all EI grades)

**Table-II:** The duration of hospitalization stratified by EI. Patients with higher EI (>2) were more likely to have prolonged hospital stays compared with those with lower EI values.

Post-operative hospital stay	No .of patients	Percentage	Edema index
Less than 5 days	11	35.5%	EI=0 = 3 (9.6%) EI<1 = 5 (16.12%) EI=1-2 = 1 (3.2%) EI>2 = 2 (6.45%)
5-10 days	13	41.9%	EI=0 = 0 (0%) EI<1 = 5 (16.12%) EI=1-2 =2 (6.45%) EI>2 = 6 (19.35%)
More than 10 days	7	22.6%	EI=0 =2 (6.45%) EI<1 = 2 (6.45%) EI=1-2 = 1 (3.2%) EI>2 = 2 (6.45%)

### Statistical analysis:

Statistical analysis was conducted to assess the association between pre-operative Edema Index (EI) and postoperative complications as well as age-related differences across edema severity groups. A chi-square test was performed to examine the relationship between EI groups (No Edema, EI < 1, EI 1–2, EI > 2) and various postoperative complications, including neurological deficits, CSF leaks, and meningitis. The association was not statistically significant, χ²(12) = 13.68, p = 0.321, suggesting that the distribution of postoperative complications did not differ meaningfully across EI categories.

Furthermore, an independent samples t-test was conducted to compare patient ages between those with EI < 1 and those with EI > 2. The mean age was slightly higher in the EI < 1 group; however, the difference was not statistically significant (t = –0.74, p = 0.466), indicating no substantial age-based disparity between patients with low versus high edema indices.

The study found that:

- There was direct relationship of edema index and the need for expansion duraplasty.
- The extent of resection was not significantly influenced by the Edema index.
- No relationship could be established between Edema index and post-operative complications.
- Longer hospitalization duration, with more extended ICU stays for patients with EI > 2.

## DISCUSSION

Meningiomas are slow growing, mostly benign tumors that originate from the meninges of the brain. Due to their slow growing nature, they can grow to a substantial size before any clinical signs appear.^12^ In addition to their mass effect, the development of peritumoral brain edema is a constant. Peritumoral Brain Edema (PTBE) increases surgical complexity by distorting anatomy, raising the risk of bleeding, and complicating tumor resection. It contributes to postoperative deficits, delayed recovery, and prolonged hospitalization. Peritumoral brain edema can be found in 70-80% of the cases.^12^,^13^ In our study 83.9% of the patients developed PTBE and 45.2% had EI>1.

Gurkanlar et al. proposed that there is a direct relationship between age and PTBE with the incidence of PTBE significantly higher in patients between 61-70 years of age.^14^ Our study found no such relationship. The incidence of PTBE in the four patients older than 60 included in our study was comparable to younger age groups. Many factors in addition to age have been discussed as possible causes influencing PTBE but results have been contrasting. Lee et el. and Ahmeti et el. found higher rates of PTBE in males as compared to females.^15^,^16^ Our study found that the severity/volume of PTBE is higher in males as compared to females. 54.54% of the males in our study had EI>2. In contrast only 20% of the females had EI>2.

Our study also found that there is a significant association of location of the tumor and the EI. 70% of the patients with EI>2 had convexity tumors followed by parasagittal meningiomas. Whereas the EI was significantly lower in patients with skull base meningiomas. Which was the same as previous studies (Frati et al.), done on the subject.^17^

We were able to achieve Grade I-II resections in 90% of patients with EI>2. Our study found no relationship between EI and the extent of tumor resection. This is in accordance with the study conducted by Ahmeti et el.^16^ We found that the extent of resection of meningiomas is more dependent on the location and the size of the tumor rather than the EI.

Our study found no relationship between higher EI and post-operative complications in contrast to the study done by Loewenstern et al.^18^ The overall complication rate was low and was similar in all EI groups. Only six patients develop neurological deficits. Only one suffered from meningitis and one from CSF leak. Our findings contrast with Loewenstern et al., who observed stronger associations between PTBE and postoperative morbidity. This difference may be explained by younger patient demographics in our cohort, the frequent use of expansion duraplasty in high-EI cases, and systematic perioperative measures such as corticosteroids and osmotic therapy, all of which may have mitigated edema-related complications despite high EI values. Prior research suggests that severe preoperative brain edema is a predictor of postoperative ischemic events but no patients included in our study had any post-operative ischemia.

A direct relationship between EI and prolonged hospital stays was found, with patients in the EI > 2 group requiring longer hospital and ICU stays. The overall hospital stays of our patients was significantly shorter as compared to previous studies.^19^,^20^ 77.4% of our patients were discharge within 10 days of surgery. This is significantly shorter as compared to the studies done by Markovic et al. and Vignes et al. who found a mean of 14.45 and 21.7 days hospital stay respectively for patients with PTBE.^19^, ^20^

Expansion duraplasty emerged as a particularly relevant intraoperative strategy in high-EI cases, where 70% of patients with EI > 2 required the procedure. In our series, duraplasty was performed in the presence of tense dura, intraoperative brain swelling, or anticipated postoperative edema. Its role extends beyond simple decompression, serving as a protective measure that may reduce secondary ischemia, venous congestion, and pressure-related neurological decline. The relatively low complication rates and shorter-than-expected hospital stays observed in our cohort may, in part, be attributable to this proactive surgical decision-making.

Overall, expansion duraplasty should not be regarded merely as a reactive maneuver but rather as a deliberate adjunct in managing high-EI meningiomas. By providing additional intracranial volume and facilitating optimal brain relaxation, it may mitigate the risks of intraoperative and postoperative swelling. In our study, expansion duraplasty was performed in 18 of 31 patients (58.1%), supporting its frequent role in high-risk cases. Although statistical analysis did not demonstrate a significant association between high EI and postoperative complications, patients with EI > 2 consistently required more complex intraoperative maneuvers and experienced prolonged hospitalization compared to those with lower EI. These findings, while limited by sample size, highlight EI as a marker of surgical complexity and perioperative resource utilization. Future studies with larger cohorts are needed to validate expansion duraplasty as a standardized strategy for mitigating edema-related morbidity and improving surgical outcomes.

Another aspect warranting consideration is the perioperative medical management of PTBE. Corticosteroids, particularly dexamethasone, are widely utilized to reduce vasogenic edema and improve preoperative neurological function and intraoperative brain relaxation.^21^ Osmotic agents such as mannitol remain valuable adjuncts in controlling raised intracranial pressure and optimizing surgical exposure.^22^ Although their use was not systematically analyzed in this study, integrating standardized steroid and osmotic therapy protocols may mitigate the impact of severe edema and improve postoperative recovery, as highlighted in existing clinical guidelines. Future prospective studies should evaluate the interaction between medical management and EI-related surgical outcomes.

### Limitations:

This study has certain limitations that warrant reconsideration. Data collection was limited to a single institution, potentially affecting the generalizability of findings to a broader patient population. Data collection was primarily done from patient’s files and medical records retrospectively, which lead to partial and inconsistent data. This study is retrospective in nature, which introduced selection bias to the study. Postoperative outcomes were assessed within a limited timeframe, preventing analysis of long-term neurological recovery and tumor recurrence. The sample size was not be large enough to detect subtle variations in outcomes across different EI groups. Also, comorbidities, steroid use, and preoperative neurological scoring were inconsistently documented in our retrospective cohort. These confounders should be systematically assessed in future prospective studies

## CONCLUSION

This study suggests that the pre-operative Edema Index (EI) and tumor location are important indicators of surgical complexity and hospitalization in supratentorial meningiomas. Although no significant associations were demonstrated with postoperative complications or extent of resection, patients with higher EI values more often required advanced intraoperative strategies (e.g. expansion duraplasty) and experienced longer hospital stays. These findings highlight EI as a promising adjunct for risk stratification rather than a validated prognostic tool. Perioperative measures such as corticosteroids, mannitol, and expansion duraplasty may further mitigate edema-related challenges.

### Recommendations:

Understanding the relationship between EI and surgical outcomes is critical for refining preoperative planning and improving patient care. Future research should explore the role of diffusion tensor imaging (DTI) and perfusion-weighted MRI in accurately assessing edema and predicting surgical outcomes. The relationship of expansion duraplasty and post-op complications should be studied on a larger scale and the role of expansion duraplasty in minimizing post-op complications in meningiomas with PTBE should be studied.

Additionally, further multi-center studies with larger patient cohorts and longer follow-ups are warranted to validate these findings and refine surgical decision-making based on EI-based risk stratification and to refine predictive models for meningioma surgery outcomes. Incorporating personalized surgical strategies for patients with high EI could significantly improve postoperative recovery and reduce complication rates. Given the retrospective design and small sample size, these results should be interpreted as exploratory. Larger, prospective multicenter studies are essential to confirm the predictive value of EI and to define standardized strategies for managing high-EI meningiomas.

### Authors’ Contributions:

**UA:** Conceived the idea, critically reviewed and supervised the study.

**NS:** Collected and analyzed the data, interpreted the results, drafted the manuscript and responsible for the accuracy of the study.

**AIS:** Literature search, Data acquisition and manuscript drafting.

**SSHS:** Literature search, critically reviewed and supervised the manuscript.

All authors have read and approved the final version of the manuscript.
